# A Cross-Sectional Observational Study Analyzing the Knowledge, Attitude, and Practice of Young Female Nursing Officers and Female Doctors About the Human Papillomavirus Vaccine

**DOI:** 10.7759/cureus.66284

**Published:** 2024-08-06

**Authors:** Mita Mandal, Subhankar Sarkar, Subrat Panda

**Affiliations:** 1 Obstetrics and Gynecology, All India Institute of Medical Sciences, Kalyani, Kalyani, IND; 2 Pediatric Nephrology, All India Institute of Medical Sciences, Kalyani, Kalyani, IND

**Keywords:** practice, attitude, knowledge, cervical cancer, hpv vaccine, human papillomavirus

## Abstract

Background

Cervical cancer (CC) is the second most common cancer among Indian women and is caused by a human papillomavirus (HPV) infection. To achieve its global commitment to the elimination of CC, India is planning to include the HPV vaccine in its national immunization program. The success of the prevention of CC mostly depends on the level of awareness and knowledge among healthcare providers about different aspects of the disease and the vaccine. We aimed to evaluate the knowledge, attitudes, and practices (KAP) regarding CC, HPV, and the HPV vaccine in first-contact young female doctors and nursing officers.

Methodology

This cross-sectional study was conducted at the All India Institute of Medical Sciences, Kalyani, between February and June 2024 among young female health workers aged between 20 and 35 years. To evaluate KAP we used a validated, self-administered questionnaire.

Results

There were a total of 204 participants, whose median age was 26 (interquartile range = 25 to 29) years; among them, 114 (55.9%) were nursing officers and 90 (44.1%) were junior doctors. Good knowledge was found among 85.5% of doctors and 70.2% of nursing officers (p < 0.01). A positive attitude was found in 81.1% of doctors and 67.5% of nursing officers (p < 0.01). The overall good practice score was low (31.3%). A higher level of education was associated with good knowledge (β = -1.16, 95% confidence interval (CI) = -1.76, -0.55, p < 0.01) and positive attitude (β = -0.53, 95% CI = -0.9, 0.16, p = 0.005) toward HPV, CC, and the HPV vaccine.

Conclusions

Our cohort showed good knowledge and attitude toward CC, HPV, and the HPV vaccine, but poor HPV vaccine uptake and practice. Therefore, health education programs focused on increasing awareness and uplifting confidence are needed to accept and recommend the HPV vaccine in developing countries like India.

## Introduction

Globally, about six lakh women were diagnosed with cervical cancer (CC), and half of them died because of the advanced stage of the disease [1]. The World Health Assembly established a goal of fewer than 4 CC cases per 100,000 women by 2030 [2]. India contributes one-fifth of the global burden of CC [3]. According to the World Health Organization, India is not adequately prepared to fight against CC [3]. The burden of CC can be effectively reduced by screening programs and human papillomavirus (HPV) vaccination. The National Family Health Survey-5 data indicated that the rate of CC screening among Indian women is merely 1.9% [4]. HPV vaccines provide more than 90% protection against CC [5]. Hence, implementing primordial prevention strategies such as mass immunization is crucial. India and 194 nations have pledged to achieve the goal of immunizing 90% of girls with the HPV vaccine before they reach 15 years of age by 2030 [3]. Despite the Drug Controller General of India’s approval of HPV vaccinations in 2008, they remain accessible only to women who can afford them [6]. As a result, uptake of the HPV vaccine remains at 2% [3]. Several factors contribute to this, including lack of awareness, limited accessibility, high cost, and misunderstanding about the potential harmful side effects of the HPV vaccine. Studies have demonstrated that there is a significant lack of understanding among the public regarding the HPV vaccine [7,8]. This study was conducted to analyze the knowledge, attitude, and practice (KAP) among female nurses and young female doctors, as they are the first line of contact with patients. The objective of this study is to identify knowledge gaps that hinder HPV vaccine acceptance.

## Materials and methods

This cross-sectional, observational study was conducted at the All India Institute of Medical Sciences (AIIMS), Kalyani, between February and June 2024. The inclusion criteria were female nursing officers and doctors (interns and junior resident doctors, both academics and non-academics) with an age range of 20 to 35 years. Those who were not willing to participate were excluded. The health professionals were informed about the nature of the study before providing written informed consent. We used a validated, self-administered questionnaire based on previous studies [9-11]. The outcome measurement of the questionnaire included (a) sociodemographic characteristics of respondents; (b) knowledge about HPV and CC; (c) knowledge about the HPV vaccine; (d) attitudes toward HPV, CC, and the HPV vaccine; (e) HPV vaccination practices; and (f) source of knowledge. The questionnaire consisted of 30 questions, of which 17 assessed knowledge, 10 assessed attitude, and three assessed practice (Appendices). The responses to the questions were of the “yes,” “no,” and “not-known/not answered” types. For each correct response, “one mark” was given, and “0” for the incorrect response.

Five experts in the relevant fields were consulted to verify the face validity of the questionnaire. A pilot test was conducted on 50 participants to check effectiveness and feasibility. To assess the relationships between the questions, exploratory factor analysis was performed. Cronbach’s alpha was calculated to determine the reliability of the questionnaire, which was 0.75 in the acceptance range of internal consistency. The questionnaire was administered separately to the participants by trained volunteers to maintain anonymity. Importance was given to avoiding any type of Googling. We assured the respondents that any part of the interview would not be shared with others. Ethical approval was obtained from the Institutional Ethics Committee (IEC) of AIIMS Kalyani (reference number: IEC/AIIMS/Kalyani/certificate/2024/087). All data were entered and cross-checked three times to avoid any errors during the data entry. All tests were conducted using a two-sided approach, and statistical significance was defined as a p-value <0.05 with a 95% confidence interval (CI). Data were expressed as mean ± SD and median interquartile range (IQR) and analyzed using the t-test. The data were analyzed using STATA version 17.0, 2021 (StataCorp., College Station, TX, USA).

## Results

A total of 215 female healthcare professionals were screened for eligibility, but 11 participants were excluded as they were not willing to share data. Hence, the data of 204 participants were analyzed. The majority of them were young, and the median age was 26 (IQR = 25 to 29) years. Among the participants, 114 (55.9%) were nursing officers, and 90 (44.1%) were junior doctors. Our respondents held a bachelor’s degree (MBBS or BSC) in nursing as their highest educational qualification. Most respondents also had wages of $1,000 (IQR = 800-11,000) per month. About 32% were unmarried, and 58.8% of the participants had never been sexually exposed. Of those who had sexual exposure, 34.8% said they had never used barrier contraception.

Knowledge

To assess knowledge, we used a total of 17 questions. Good knowledge was classified as having a score of ≥60%, which is ≥10; any score <10 denoted inadequate knowledge. Good knowledge was found in 76.9% of participants, with a mean knowledge score of 11 ± 1.9. Among them, 85.5% were doctors and 70.2% were nursing officers (p < 0.01). Table 1 summarizes the good knowledge, positive attitude, and good practices toward HPV infection, CC, and the HPV vaccine of the respondents. The majority of doctors and nursing officers responded correctly to some points of knowledge questionnaires, such as HPV can cause CC (98.6%), genital warts (86%), HPV is sexually transmitted (90%), and sexually active women can be infected at any time (74%). They also knew that the HPV vaccine was not 100% protective (72.9%), they needed three doses of the HPV vaccine to develop immunity (72.9%), and the HPV infection was not likely to be completely cured by clinical treatment (68.6%). Lack of knowledge was found in some areas: only 17.7% knew that a single dose of HPV is ineffective and available for use in India, 53.3% knew that there are two types of HPV vaccines, but only 31.8% knew about “Cervavac,” and 16.6% knew whether the cost of the vaccine was reimbursable or not. The mean score of knowledge was significantly higher in doctors (11.8 ± 1.9) than in nursing officers (10.4 ± 1.8) (p < 0.01).

**Table 1:** Good knowledge, positive attitude, and good practice toward HPV infection, CC, and HPV vaccination of the respondents. CC = cervical cancer; HPV = human papillomavirus

Questions	Nursing officers (%)	Doctors (%)	P-value
Knowledge			
Cervical cancer is mainly caused by HPV infections	97.3	100	0.12
Certain types of HPV can cause genital warts	79.8	92.2	0.01
HPV is sexually transmitted	86.8	93.3	0.13
HPV infection is not likely completely cures by clinical treatment	59.6	77.7	0.005
Most sexually active women are infected by HPV at some point in their lives	64	84.4	0.001
The most important way of screening for cervical cancer			
Screening for all females	40	58	0.03
Vaccination for all girls	52.2	52	0.98
Screening all mothers and vaccinating their daughters	30	21	0.81
Safe sexual practice	16.6	7	0.76
HPV vaccines cannot 100% prevent cervical cancer	73.6	72.2	0.81
It is recommended that females should receive all three shots of HPV vaccination within a six-month period	69.2	76.6	0.24
A single dose of the vaccine is effective	16.6	18.8	0.068
Names of two HPV vaccines	33.3	73.3	<0.01
Knows about Cervavac	19.2	44.4	0.001
Knows that Cervavac is a costly vaccine	13.1	24.4	0.03
Regular screening for cervical cancer is done after vaccination	54.3	71.1	0.03
The cost of the HPV vaccine is reimbursable	21	12.2	0.12
Attitude			
I may be infected by HPV in the future	74.5	78.8	0.47
I may have cervical cancer	66.6	76.6	0.05
Cervical cancer is a severe disease	85.9	93.9	0.18
HPV vaccine can effectively prevent cervical cancer	83.3	76.6	0.32
HPV is a costly vaccine	74.5	75.5	0.55
It is not easy to find a place to receive the HPV vaccine	58.7	57.7	0.42
I am afraid of the side effects of HPV vaccination	61.5	67.8	0.97
I am afraid of being perceived as sexually active if I receive the HPV vaccine	72	61.2	0.17
Parents affect my decision as to whether or not to receive the HPV vaccine	28.8	17.5	0.008
The doctor’s recommendation affects my decision as to whether or not to receive the HPV vaccine	45.6	60	0.01
Practice			
Received at least one shot of the HPV vaccine	13.1	5.5	0.07
If the cost is reduced, I will definitely receive it	64.9	61.1	0.56
I will do screening regularly and not take the vaccine	41.2	35.5	0.41

Attitude and practice

A positive attitude was considered if the individual scored ≥60%, which is ≥6 out of 10. The overall mean score for attitude was 5.6 ± 1.3. About 73.5% of participants showed a positive attitude toward the HPV vaccine. Among them, 67.5% were nursing officers, and 81.1% were doctors. The mean score for attitude was significantly higher in doctors (5.9 ± 1.2) than in nursing officers (5.4 ± 1.3) (p < 0.01). The majority agreed that they might get infected by HPV in the future (76.6%), might have CC (71.6%), that CC is a severe disease (89.9%), and that the HPV vaccine can effectively prevent CC (79.9%). Moreover, the majority denied that HPV is a costly vaccine (75%), it is difficult to find a place to get the HPV vaccine (58.2%), they are afraid of the side effects of HPV vaccination (64.7%), they are perceived as sexually active after HPV vaccination (66.6%), and their decision to take the vaccine was not affected by parents (76.9%) or doctor recommendation (47.2%). A good practice score of ≥60% (≥2 out of 3) was found in 31.3%, and the overall mean practice score was 1.1 ± 0.7. The mean practice score was higher in nursing officers (1.2 ± 0.7) than in doctors (1.0 ± 0.7) (p = 0.04). Only 9.3% received at least one dose of the HPV vaccine, and 38.3% would prefer to do screening rather than take the vaccine, but the majority (63%) agreed to take the vaccine if the cost was reduced.

The bivariate and multivariate logistic regression analyses of several factors contributing to good knowledge and attitude are summarized in Table 2. A higher level of education (β = -1.16, 95% CI = -1.76, -0.55, p < 0.01) was associated with good knowledge of HPV, CC, and the HPV vaccine. Higher educational level (β = -0.53, 95% CI = -0.9, 0.16, p = 0.005) and income (β = -0.02, 95% CI = -0.03, -0.004, p = 0.007) were associated with a positive attitude toward HPV, CC, and the HPV vaccine. In bivariate analysis, none of the factors were significantly associated with good practice. The principal sources of knowledge for participants were the Internet (88%), books (81%), and magazines (68%).

**Table 2:** Bivariate and multivariate logistic regression analysis of factors contributing to good knowledge and attitude toward HPV, CC, and the HPV vaccine. CC = cervical cancer; HPV = human papillomavirus; CI = confidence interval

Variable	Knowledge				Attitude			
	Unadjusted β, 95% CI	P-value	Adjusted β, 95% CI	P-value	Unadjusted β, 95% CI	P-value	Adjusted β, 95% CI	P-value
Age	-0.14 (0.2, 0.05)	0.001	-0.05 (-0.14, 0.04)	0.28	-0.02 (0.07, 0.02)	0.59	-0.05 (-0.14, 0.04)	0.28
Education	-1.3 (-1.8, -0.8)	<0.01	-1.2 (-1.8. -0.55)	<0.01	-1.3 (-1.8, -0.8)	<0.01	-1.2 (-1.8, -0.55)	<0.01
Income	0.01 (-0.03, -0.002)	0.08	-	-	0.01 (-0.03, -0.002)	0.08	-	-
Prior sex exposure	0.39 (-0.17, 0.95)	0.17	-	-	0.39 (-0.17, 0.95)	0.17	-	-

## Discussion

To our knowledge, this study is the first in India to investigate the KAP of HPV, CC, and the HPV vaccine among young female health workers. Adequate knowledge and attitude of healthcare workers toward HPV infection, CC, and HPV vaccination can readily improve their practices and ensure that accurate information is delivered to patients. Junior doctors and nursing officers are the first-contact health workers in the community, especially in rural India where older and more experienced as well as specialist doctors are not available. In India, the HPV vaccine is only approved for adolescent girls and women [12]. We chose young female health workers in our study to assess their KAP for the HPV vaccine and CC as they could have recently experienced the HPV vaccine for their own health, which mirrors the HPV vaccine and CC awareness in society.

Our results show that the majority of our health workers had good knowledge (76.9%) and a positive attitude (73.5%), but their practice score was low (31.3%), which indicates a gap in delivering care. Despite good knowledge and a positive attitude, only 9.3% of participants received the HPV vaccine, which reflects the gap between knowledge and acceptance of the vaccine. Although this value is slightly better than the general population (2%) but far away from the target level [3], this finding warrants the need for root-cause analysis to improve practice. Several studies in India have indicated poor knowledge about HPV infection, cervical cancer, and the vaccine. Recently, in a meta-analysis, Debkumar et al. reported that the pooled prevalence of knowledge, positive attitude to uptake, and coverage of HPV vaccines in India were 0.22 (CI = 0.14-0.31, I^2^ = 99.5%), 0.45 (CI = 0.33-0.57, I^2^ = 100%), and 0.04 (CI = 0.02-0.07, I^2^ = 96%), respectively [13]. Similar to our study, Chawla et al. reported that about 84-90% of healthcare providers were aware of CC and HPV, but there is a lack of awareness among paramedical staff [14]. Dabash et al. found a gap in the knowledge of the causative nature of HPV and CC, but there is a lack of understanding of the natural history of CC and its preventable nature through screening and vaccination [15]. Similar cross-sectional studies were conducted to assess KAP regarding CC, HPV, and HPV vaccines among healthcare providers in Bangladesh, Malaysia, and South West Nigeria, which showed quite low knowledge levels (43.29%, 62.9%, and 67.1%, respectively) [16-18]. Similar to our study, good vaccine practice was low in Bangladesh (11.8%) [16] and China (2.02%) [19]. Mohamed et al. assessed the KAP of CC screening and HPV vaccination in a cohort of 250 Egyptian gynecologists. They found that 45% had adequate knowledge, 57% had a positive attitude, and only 45% of participants had prescribed the HPV vaccine at least once [20].

Since 2008, commercially, two vaccines have been available in India: a quadrivalent (Gardasil) and a bivalent (Cervarix) vaccine. The indigenously developed quadrivalent HPV vaccine, commercially called Cervavac, was launched in India in September 2022 [4]. Recently, the Government of India stated its intention to incorporate HPV into the nation’s immunization program to prevent CC among girls aged 9-14 years [21]. Cervavac seems to be a potential weapon to fight against CC for Indian women [22]. Our study shows poor knowledge among doctors and nurses about Cervavac and its cost. Only 19% of nurses and 44% of doctors were aware of the Cervavac vaccine, and 13% of nurses and 24% of doctors knew about the cost of the same vaccine. This finding mandates the need for training and education among doctors and nurses. Our study found that only 5.5% of doctors and 13% of nurses received at least one dose of the HPV vaccine, but 63% agreed that they would take the vaccine if the cost of the vaccine was reduced. This indicates that our cohort was not fully aware of the cost of the HPV vaccine and its protection benefits.

The present study has several limitations such as a small sample size, single-center and cross-sectional design, and limited follow-up. However, despite its size, this study represents a homogenous cohort of young female front-line health workers from a newly developed tertiary care hospital in eastern India.

## Conclusions

KAP surveys are popular in healthcare because they provide useful information and appear easy to design and execute. As India is preparing for its Indigenous vaccination program, this study is important as its target subjects are first-contact physicians and nurses. Our cohort showed good knowledge and attitude but poor uptake and practice toward CC, HPV, and the HPV vaccine. Therefore, health education programs focus on increasing awareness and uplifting confidence to accept and recommend the HPV vaccine, which is much needed. Moreover, the government should endeavor to include the HPV vaccine in the National Immunization Program.

## Appendices

**Table 3:** Study questionnaire.

Name				
Age (years)				
Maximum level of education				
Average family income per month				
Marital status				
Have sex experience				
Type of contraception used				
Answer all questions below				
Questions		Mark () appropriate answer		
“Cervical cancer is mainly caused by HPV infections”		Yes	No	Don’t know
“Human papillomavirus (HPV) is sexually transmitted”				
“Certain types of HPV can cause genital warts”				
“HPV is sexually transmitted”				
“HPV infection is not likely to be completely cured by clinical treatment”				
“The most important way of screening for cervical cancer” Screening for all females; Vaccination for all girls; Screening all mothers and vaccinating their daughters; Safe sexual practice				
“HPV vaccines cannot 100 percent prevent cervical cancer”				
“It is recommended that female should receive all 3 shots of HPV vaccination within a 6-month period”				
“A single-dose vaccine is effective”				
“Names of two HPV vaccines”				
“Know about Cervavac”				
“Cervavac is a costly vaccine”				
“Regular screening of cervical cancer done after vaccination”				
“Cost of HPV is reimbursable				
“I may be infected by HPV in the future”				
“I may have cervical cancer”				
“Cervical cancer is a severe disease”				
“HPV vaccine can effectively prevent cervical cancer”				
“HPV is a costly vaccine”				
“It is not easy to find a place to receive to HPV vaccine”				
“I am afraid of the side effects of HPV vaccination”				
“I am afraid of being perceived as sexually active if I received HPV vaccine				
“Parents affect my decision as to whether or not to receive HPV vaccine”				
“Doctor’s recommendation affects my decision as to whether or not to receive HPV vaccine”				
“Received at least one shot of HPV vaccine”				
“If the cost came down, I will definitely receive it”				
“I will do screening regularly not take the vaccine”				
Please mark () the source of your knowledge				
Television advertisements		Schools		
Internet		Peers		
Newspapers		Parents		
Magazines		Outdoor advertisements		
Books		Family doctors		
Other than above then mention here:				

## References

[1] Bray F, Ferlay J, Soerjomataram I, Siegel RL, Torre LA, Jemal A (2018). Global cancer statistics 2018: GLOBOCAN estimates of incidence and mortality worldwide for 36 cancers in 185 countries. CA Cancer J Clin.

[2] (2024). World Health Assembly. Global strategy to accelerate the elimination of cervical cancer as a public health problem and its associated goals and targets for the period 2020-2030. Internet.

[3] (2024). Ministry of Health and Family Welfare, Government of India. National Family Health Survey-5, 2021. National Family Health Survey-5.

[4] Gopika MG, Prabhu PR, Thulaseedharan JV (2022). Status of cancer screening in India: an alarm signal from the National Family Health Survey (NFHS-5). J Family Med Prim Care.

[5] Deleré Y, Wichmann O, Klug SJ, van der Sande M, Terhardt M, Zepp F, Harder T (2014). The efficacy and duration of vaccine protection against human papillomavirus: a systematic review and meta-analysis. Dtsch Arztebl Int.

[6] Das BC, Hussain S, Nasare V, Bharadwaj M (2008). Prospects and prejudices of human papillomavirus vaccines in India. Vaccine.

[7] Mehta S, Rajaram S, Goel G, Goel N (2013). Awareness about human papilloma virus and its vaccine among medical students. Indian J Community Med.

[8] Kamini Kamini, S S, Bhimarasetty DM (2016). Awareness about human papilloma virus vaccine among medical students. Asian J Med Sci.

[9] Rashid S, Labani S, Das BC (2016). Knowledge, awareness and attitude on HPV, HPV vaccine and cervical cancer among the college students in India. PLoS One.

[10] Yacouti A, Elkhoudri N, El Got A (2022). Awareness, attitudes and acceptability of the HPV vaccine among female university students in Morocco. PLoS One.

[11] Kim HW, Lee EJ, Lee YJ, Kim SY, Jin YJ, Kim Y, Lee JL (2022). Knowledge, attitudes, and perceptions associated with HPV vaccination among female Korean and Chinese university students. BMC Womens Health.

[12] Vashishtha VM, Choudhury P, Kalra A (2014). Indian Academy of Pediatrics (IAP) recommended immunization schedule for children aged 0 through 18 years--India, 2014 and updates on immunization. Indian Pediatr.

[13] Aggarwal S, Agarwal P, Singh AK (2023). Human papilloma virus vaccines: a comprehensive narrative review. Cancer Treat Res Commun.

[14] Chawla PC, Chawla A, Chaudhary S (2016). Knowledge, attitude & practice on human papillomavirus vaccination: a cross-sectional study among healthcare providers. Indian J Med Res.

[15] Dabash R, Vajpayee J, Jacob M, Dzuba I, Lal N, Bradley J, Prasad LB (2005). A strategic assessment of cervical cancer prevention and treatment services in 3 districts of Uttar Pradesh, India. Reprod Health.

[16] Chowdhury S, Ara R, Roy S (2022). Knowledge, attitude, and practices regarding human papillomavirus and its' vaccination among the young medical professionals and students of Bangladesh. Clin Exp Vaccine Res.

[17] Rashwan HH, Saat NZ, Abd Manan DN (2012). Knowledge, attitude and practice of malaysian medical and pharmacy students towards human papillomavirus vaccination. Asian Pac J Cancer Prev.

[18] Adejuyigbe FF, Balogun MR, Sekoni AO, Adegbola AA (2015). Cervical cancer and human papilloma virus knowledge and acceptance of vaccination among medical students in Southwest Nigeria. Afr J Reprod Health.

[19] An J, Liu Y, Ma Y (2024). Real-world data of China: analysis of HPV vaccine coverage and post-vaccination adverse reaction monitoring in Western Chinese provinces from 2018 to 2021. Hum Vaccin Immunother.

[20] Mohamed ML, Tawfik AM, Mohammed GF, Elotla SF (2022). Knowledge, attitude, and practice of cervical cancer screening, and HPV vaccination: a cross-sectional study among obstetricians and gynecologists in Egypt. Matern Child Health J.

[21] (2024). Ministry of Finance, Government of India. Nari Shakti Takes Center Stage. Union Finance Minister announces vaccination to prevent cervical cancer. https://pib.gov.in/PressReleaseIframePage.aspx?PRID=2001172.

[22] Gupta S, Kumar P, Das BC (2023). Challenges and opportunities to making Indian women cervical cancer free. Indian J Med Res.

